# Synthesis of [^2^H_5_]baricitinib via [^2^H_5_]ethanesulfonyl chloride

**DOI:** 10.1002/jlcr.3969

**Published:** 2022-03-24

**Authors:** Ross D. Jansen‐van Vuuren, Rahul Vohra

**Affiliations:** ^1^ Department of Chemistry Queen's University Kingston Ontario Canada; ^2^ Faculty of Chemistry and Chemical Technology University of Ljubljana Ljubljana Slovenia; ^3^ Department of Chemistry and Business Development Sussex‐Research Lab Inc. Ottawa Ontario Canada

**Keywords:** baricitinib, COVID‐19, deuteration, deuterium‐labelled, isotopologue, SARS‐CoV‐2

## Abstract

Baricitinib, typically applied as a treatment for rheumatoid arthritis, has recently attracted the attention of clinicians and researchers as a potential treatment for COVID‐19. Naturally, there has been a need for the preparation of the isotope‐labelled analogue of baricitinib to probe the pharmacokinetics of baricitinib in this new role. As such, we have developed a simple synthetic route to deuterated [^2^H_5_]baricitinib, facilitating its formation over four steps and in a 29% overall yield based on starting [^2^H_5_]ethanethiol (19% if we start with [^2^H_5_]bromoethane instead). A critical component of the overall process involves the synthesis of [^2^H_5_]ethanesulfonyl chloride, and we describe in detail the two routes that were explored to optimize this step.

## Graphical abstract

Recently, baricitinib has attracted interest for its potential role as a therapeutic agent in the treatment of COVID‐19, creating a need to develop a synthetic route to its deuterium‐labelled analogue for use as a mass spectrum internal standard, since the isotopologue is typically somewhat costly. This paper presents a new synthetic route to [^2^H_5_]baricitinib from [^2^H_5_]ethanesulfonyl chloride. Since the preparation and isolation of [^2^H_5_]ethanesulfonyl chloride form a major challenge, these comprise a large focus of the overall work.

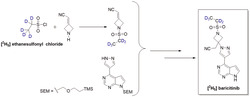

## 1 INTRODUCTION

**FIGURE 1:**
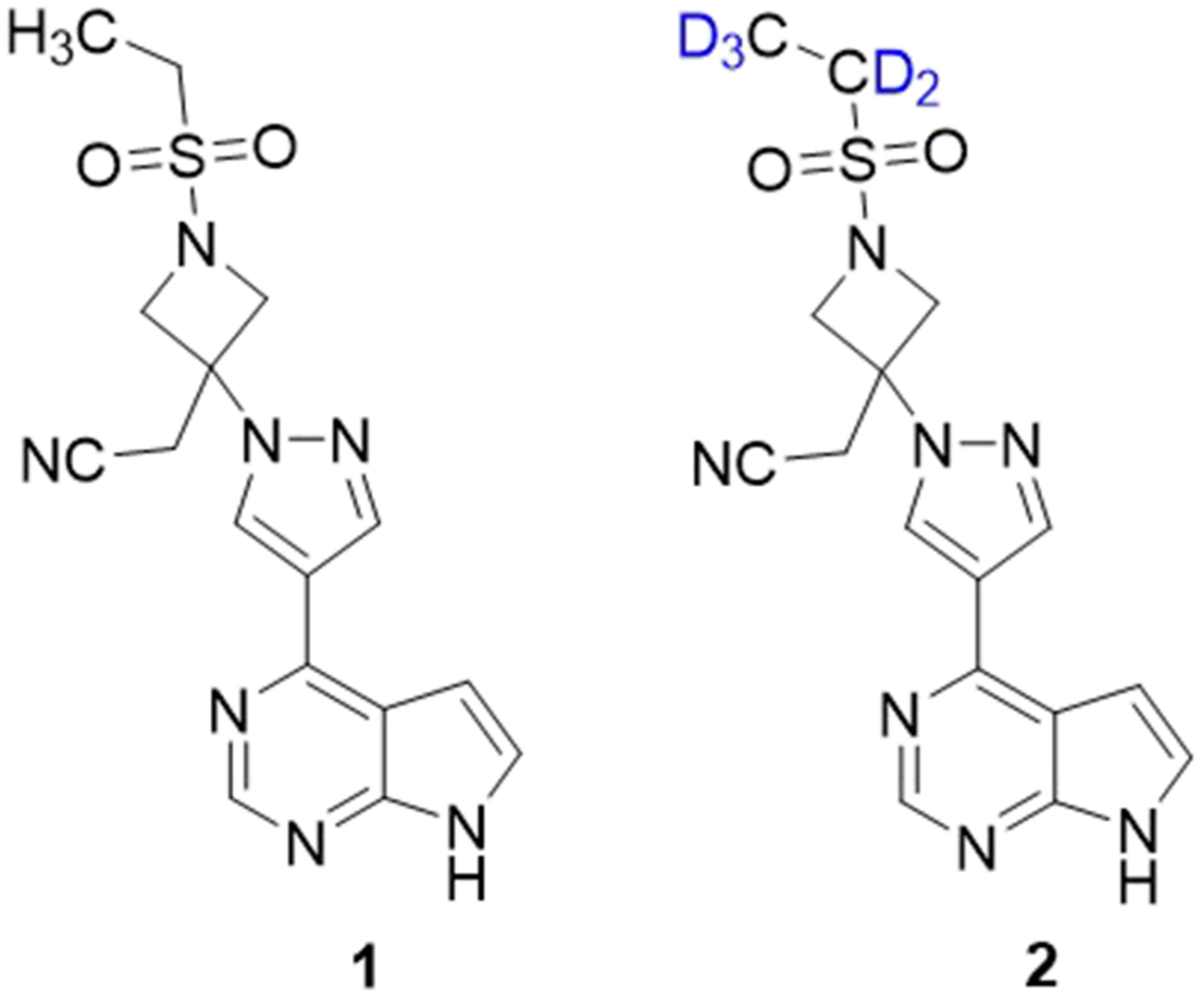
Chemical structures of baricitinib (**1**) and [^2^H_5_]baricitinib (**2**)

Baricitinib (**1**, Figure 1), a Janus kinase (JAK) inhibitor typically used in the treatment of rheumatoid arthritis, has recently garnered interest for its potential application as an antiviral treatment for severe acute respiratory syndrome coronavirus 2 (SARS‐CoV‐2).^1^, ^2^, ^3^, ^4^, ^5^ To probe the utility of baricitinib in treating COVID‐19, isotope‐labelled baricitinib would be beneficial for use as a mass spectrum internal standard in bioanalytical assays to quantify the concentration of the drug in biological samples, as has been shown with other drugs.^6^, ^7^ Although the synthesis of deuterium‐labelled baricitnib, specifically [^2^H_5_]baricitinib, has been published, this was prophetic and involved the use of noxious gaseous reagents.^8^ Thus, we were motivated to develop an alternative synthetic route to [^2^H_5_]baricitinib (**2**, Figure 1). Because we chose to insert the deuterium on the ethanesulfonyl component of **2**, a major component of the research involved finding a suitable route to the necessary precursor: a deuterated form of ethanesulfonyl chloride. The results from this exploration are presented in this work.

## 2 RESULTS AND DISCUSSION

**SCHEME 1:**
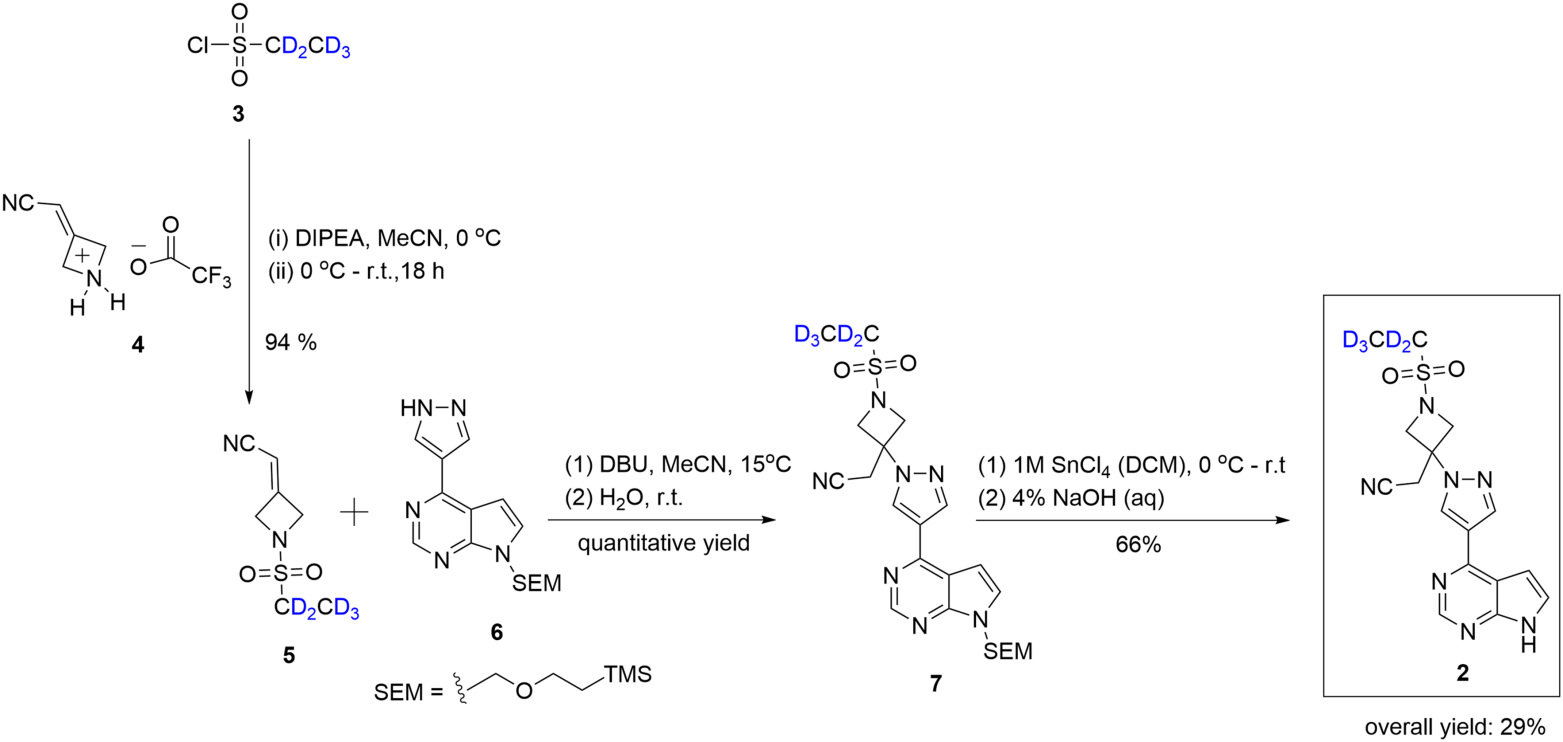
Synthetic route to [^2^H_5_]baricitinib (**2**) commencing from [^2^H_5_]ethanesulfonylchloride (**3**)

We chose to insert the deuterium on the ethanesulfonyl component via [^2^H_5_]ethanesulfonyl chloride (**3**) after rationalizing that **3** could be converted to the stable intermediate **5** upon reaction with **4**. Compound **5** could then be converted to the desired product **2** in a further two steps (reaction of **5** with commercially available **6** to form intermediate **7**, followed by trimethylsilylethoxymethyl [SEM] deprotection of **7** to provide **2**) (Scheme 1). Our synthetic approach was derived from the original route to non‐deuterated baricitinib developed by Rodgers et al.^9^, ^10^

**SCHEME 2:**
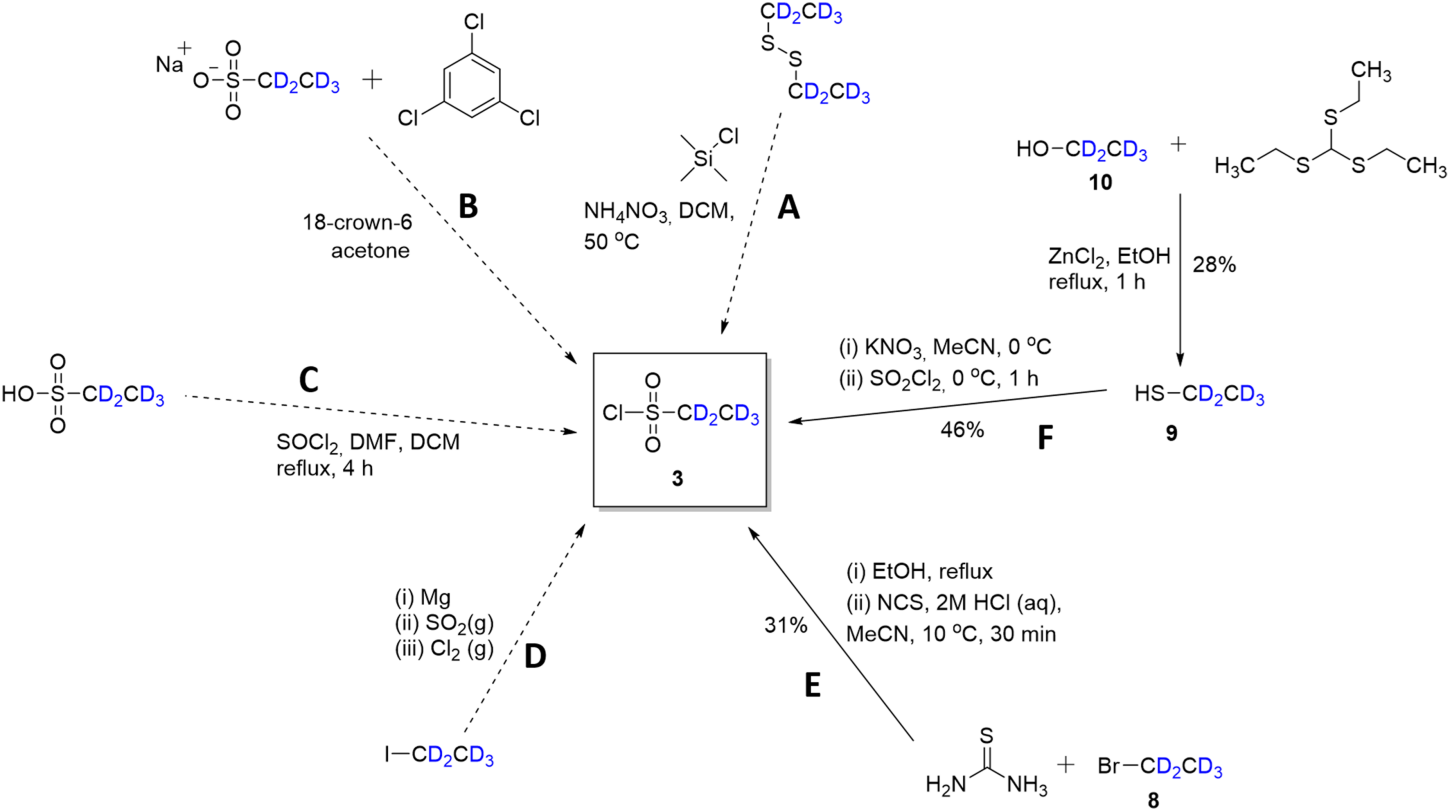
Various routes (**A**–**F**) to intermediate **3**

The main challenge associated with this approach largely lay in the first step: the preparation and isolation of **3**, as this compound is not available commercially. A preliminary literature search revealed several routes to non‐deuterated ethanesulfonyl chloride. However, a prerequisite for selection of a route for the preparation of **3** was the availability of deuterated substrates. Thus, routes commencing with diethyl disulfide^11^, ^12^, ^13^, ^14^ (route **A**), sodium ethanesulfonate^15^ (route **B**) and ethanesulfonic acid^16^ (route **C**) (Scheme 2) were *not* selected as none of these substrates are commercially available in the deuterated form. Route **D**, a prophetic route from the patent literature,^8^ involved the use of noxious gases SO_2_ and Cl_2_, so was also not attempted. Instead, we chose to explore routes **E** and **F**, which used [^2^H_5_]bromoethane (**8**) and [^2^H_5_]ethanethiol (**9**), respectively.

Preparation of **3** using route **E** is based on the procedure reported by Yang and Xu.^17^ However, as our highest yield utilizing this approach was only 31%, we attempted the synthesis of **3** via route **F**, based on a procedure developed by Park et al.,^18^ commencing with [^2^H_5_]ethanethiol (**9**).

Starting with commercial [^2^H_5_]ethanethiol (**9**), an average yield of 46% of **3** was obtained. However, given the very high cost of **9**, we also explored the possibility of preparing it from [^2^H_5_]ethanol (**10**) via an interchange reaction with commercially available tris(ethylthio)methane, which has previously been published for the preparation of non‐labelled ethanethiol.^19^ Unfortunately, this reaction (**10** → **9**) only provided **9** in relatively low yield (28%). Nevertheless, we were able to prepare sufficient of **3** using the two routes to proceed to the next step of the sequence.

Coupling of **3** with freshly prepared **4**, obtained by *N*‐Boc deprotection of *tert*‐butyl 3(cyanomethylene)azetidine‐1‐carboxylate,^8^ resulted in the formation of **5** in a high yield (94%), without the need for further purification. The following step, a nucleophilic addition reaction between compound **4** and commercially available 4‐(1*H*‐pyrazol‐4‐yl)‐7‐((2‐(trimethylsilyl)ethoxy)methyl)‐7*H*‐pyrrolo[2,3‐*d*]pyrimidine (**6**), based on the procedure published in the patent literature,^20^ proceeded in the presence of 1,8‐diazabicyclo[5.4.0]undec‐7‐ene (DBU) at r.t., resulting in the formation of **7** in quantitative yield. SEM deprotection of **7** was attempted unsuccessfully with LiBF_4_/MeCN,^21^, ^22^ TFA/ethylene diamine^23^ and BF_3_·Et_2_O,^24^ before complete deprotection was achieved by reaction with a 1 M solution of tin(IV) chloride at room temperature followed by a basic workup at 0°C^25^ forming **2** in 66% yield. This approach was employed thenceforth. The yield of the entire reaction sequence was a reasonable 29%.

## 3 CONCLUSION

In this paper, we report the synthesis of [^2^H_5_]baricitinib in an overall 29% yield. Our synthetic pathway was based on the route to non‐deuterated baricitinib developed by Rodgers et al.^10^ Several routes to the important non‐commercial intermediate [^2^H_5_]ethanesulfonyl chloride were considered; however, only two were explored experimentally, and we found that the route commencing from [^2^H_5_]bromoethane was slightly lower yielding (31%) compared with when the synthetic sequence commenced with [^2^H_5_]ethanethiol (46%). These synthetic routes provide an opportunity to prepare [^2^H_5_]baricitinib, circumventing the need to purchase it. [^2^H_5_]Baricitinib is significant as an internal reference standard or potentially a COVID‐19 therapeutic with improved efficacy compared with the non‐deuterated analogue. To evaluate the latter, metabolic profiling studies of both baricitinib and [^2^H_5_]baricitinib must be carried out.

## 4 EXPERIMENTAL

^1^H NMR (400 and 500 MHz) and ^13^C NMR (101 and 126 MHz) spectra were recorded on Bruker AV‐400 and NEO‐500 instruments in CDCl_3_ or DMSO‐*d*_6_ (as indicated). The chemical shifts are reported in δ (ppm) relative to residual CHCl_3_ or DMSO, respectively, as the internal standard. High‐resolution mass spectra (HRMS) were recorded on a Micromass 70‐250S double focusing mass spectrometer.

### 4.1 Materials

All dry solvents used were purified under an argon atmosphere according to Armarego and Chai^26^ or purchased from commercial sources. *N*‐Chlorosuccinimide (NCS) was recrystallized from glacial acetic acid. All commodity chemicals were purchased from commercial sources and used without further purification. *tert*‐Butyl 3‐(cyanomethylene)azetidine‐1‐carboxylate was obtained from Ambeed (A124948). 4‐(1*H*‐Pyrazol‐4‐yl)‐7‐((2‐(trimethylsilyl)ethoxy)‐methyl)‐7*H*‐pyrrolo[2,3‐*d*]pyrimidine was obtained from Combi‐Blocks (ST‐0174). Tris(ethylthio)methane was obtained from TCI Chemicals (T3140). ZnCl_2_ (anhydrous, free‐flowing, Redi‐Dri™, reagent grade, ≥98%), boron trifluoride diethyl etherate (BF_3_·Et_2_O), lithium tetrafluoroborate (LiBF_4_), ethylene diamine, trifluoroacetic acid (TFA), sulfuryl chloride (SO_2_Cl_2_) and methyl *tert*‐butyl ether (MTBE) were obtained from Aldrich. Deuterated chemicals were obtained from CDN Isotopes. Trifluoroacetic acid (TFA), *N*,*N*‐diisopropylethylamine (DIPEA) / Hünig's base, DBU and anhydrous acetonitrile (MeCN) were obtained from Alfa Aesar and used without further purification.

### 4.2 Experimental procedures

#### 4.2.1 [^*2*^H_5_]Ethanesulfonyl chloride (**3**)

Route **E** ^17^: [^2^H_5_]Bromoethane (5.00 g, 48.9 mmol) and thiourea (3.33 g, 48.9 mmol) were refluxed in anhydrous ethanol (44 ml) for 1 h. After cooling the reaction mixture to r.t., the ethanol was removed *in vacuo*, and the residual white oil was slowly added to a stirred mixture of NCS (29.3 g, 219.3 mmol) and 2 M HCl (aq) (22 ml) in MeCN (56 ml) at 10°C, which gradually became a bright yellow solution in the process. This new reaction mixture was stirred at 10°C for a further 30 min before Et_2_O (50 ml) was added and the organic components extracted. The organic layer was then concentrated to an orange oil, which was rapidly passed through a silica plug (eluent: hexanes → 1:4 [EtOAc:hexanes] → 2:3 EtOAc:hexanes; co‐spot with commercially available non‐deuterated ethanesulfonyl chloride: *R*_*f*_ = 0.75 in 2:3 EtOAc:hexanes; for visualization: stain by spraying TLC plate with a 10% solution of NaI in acetone^27^), enabling the isolation of **3** as a pale‐yellow liquid (1.82 g, 31%), which was immediately used in the next step to form compound **5**. Route **F** ^18^, ^19^: A mixture of tris(ethylthio)methane (4.7 ml, 25 mmol) in [^2^H_5_]ethanol (5 g, 100 mmol) was refluxed with anhydrous ZnCl_2_ (102 mg, 0.75 mmol) for 48 h before [^2^H_5_]ethanethiol (**9**) (1.88 g, 28%) was distilled off (oil bath set to 50°C); Ar balloon was attached to condenser to ensure reasonably constant internal pressure of ~1 bar. In order to contain the stench of the [^2^H_5_]ethanethiol, the flask containing the distillate must instantly be capped and transferred to the refrigerator for storage under Ar (or used immediately in the next step). [^2^H_5_]Ethanethiol (2 g, 29.8 mmol) was added to anhydrous MeCN (100 ml) under Ar at 0°C before freshly distilled sulfuryl chloride (SO_2_Cl_2_) (6 ml, 74.5 mmol) and anhydrous KNO_3_ (7.53 g, 74.5 mmol) were rapidly added, and the reaction mixture was stirred for 1 h at 0°C. The mixture was then quenched by the dropwise addition of saturated NaHCO_3_ (aq) (added until pH = 8) after which the organic component was extracted with Et_2_O (3 × 40 ml), washed with brine (50 ml) and dried over anhydrous MgSO_4_. Filtration followed by concentration of the filtrate *in vacuo* resulted in the isolation of 1.86 g of **3** (46%) as a pale‐yellow liquid, taken immediately through to the next step.

#### 4.2.2 [^*2*^H_5_]2‐(1‐((Ethyl)sulfonyl)azetidin‐3‐ylidene)acetonitrile (**5**)

TFA (28 ml, 360 mmol) was added dropwise to a solution of *tert*‐butyl 3‐(cyanomethylene)azetidine‐1‐carboxylate (3.5 g, 18 mmol) in anhydrous DCM (250 ml), which was stirred at r.t. for 5 h before being reduced to dryness *in vacuo*; 2.2 g of **4**, an amorphous white solid, was obtained and immediately suspended in 211 ml of anhydrous acetonitrile under an inert atmosphere at 0°C. DIPEA (11.7 ml, 67.4 mmol) was added dropwise, ensuring that a temperature of <5°C was maintained throughout. This was followed by the dropwise addition of **3** (1.8 g, 13.5 mmol), also ensuring that a temperature of <5°C was maintained throughout. The reaction mixture was allowed to warm to room temperature before being left to stir at this temperature for 16 h. The reaction mixture was concentrated *in vacuo*, and the resultant residue (a red/orange oil) was diluted with DCM (100 ml) before being washed with brine (100 ml). The combined organic fractions were dried over anhydrous Na_2_SO_4_ before the solvent was removed *in vacuo*. The crude material was purified by flash chromatography over silica using hexane/ethyl acetate (60/40–20/80) as eluent, to obtain 1.94 g (94%) of **5** as a yellow oil, which forms a white amorphous solid when left to stand: ^1^H NMR (400 MHz, CDCl_3_) δ ppm 5.38 (s, 1H), 4.72–4.62 (m, 4H); ^13^C NMR (101 MHz, CDCl_3_) δ ppm 155.3, 113.9, 94.6, 58.9, 58.6 (should only be 4). HRMS (ESI‐TOF) *m/z*: [M + H]^+^ calcd for C_7_H_5_D_5_N_2_O_2_S: 192.08411; found 192.08496.

#### 4.2.3 [^*2*^H_5_]2‐(1‐((Ethyl)sulfonyl)‐3‐(4‐(7‐((2‐(trimethylsilyl)ethoxy)methyl)‐7*H*‐pyrrolo[2,3‐*d*]pyrimidin‐4‐yl)‐1*H*‐pyrazol‐1‐yl)azetidin‐3‐yl)acetonitrile (**7**)

To a suspension of **5** (0.5 g, 2.61 mmol) and 4‐(1*H*‐pyrazol‐4‐yl)‐7‐((2‐(trimethylsilyl)ethoxy)‐methyl)‐7*H*‐pyrrolo[2,3‐*d*]pyrimidine (6.87 mg, 2.18 mmol) in anhydrous acetonitrile (6.83 ml) was added DBU (0.39 ml, 2.61 mmol) dropwise while keeping the temperature between 15°C and 25°C. After the addition of DBU, the reaction mixture was stirred for 30 min at r.t. until a precipitate formed. The reaction mixture was then allowed to stir for a further 16 h before being quenched with distilled water (10 ml) and stirred for a further 30 min at r.t. prior to filtering. The solid residue (**7**) was washed with water (50 ml) followed by MTBE (50 ml) and left to dry under ambient conditions before being collected (0.96 g, quantitative yield): ^1^H NMR (500 MHz, CDCl_3_) δ ppm 8.88 (s, 1H), 8.48 (s, 1H), 8.37 (s, 1H), 7.44 (d, *J* = 4 Hz, 1H), 6.81 (d, *J* = 3.5 Hz, 1H), 5.70 (s, 2H), 4.66 (d, *J* = 9.5 Hz, 2H), 4.28 (d, *J* = 9.5 Hz, 2H), 3.57 (t, *J* = 8 Hz, *J* = 8.25 Hz, 2H), 3.43 (s, 2H), 0.94 (t, *J* = 8.5 Hz, *J* = 8.25 Hz, 2H), 0.04 (s, 9H); ^13^C NMR (126 MHz, CDCl_3_) δ ppm 152.3, 151.8, 150.1, 140.8, 128.8, 128.1, 123.4, 115.0, 114.4, 100.6, 72.8, 66.6, 58.9, 56.1, 27.7, 17.7, 1.5. HRMS (ESI‐TOF) *m/z*: [M + H]^+^ calcd for C_22_H_27_D_5_O_3_N_7_SSi 507.23504; found 507.23650.

#### 4.2.4 [^*2*^H_5_]Baricitinib (**2**)

To an ice‐cold (0°C) solution of **7** (850 mg, 1.68 mmol) in anhydrous DCM (50 ml) was added a solution of SnCl_4_ (23 ml, 1 M in DCM) over 30 min. This reaction mixture was stirred at 0°C before being left to warm to r.t. until the deprotection was complete (progress tracked using TLC). The reaction mixture was then cooled to 0°C and quenched with 4% NaOH (added until pH = 8) before being left to stir for a further 15 min. The organic fraction was then separated, washed with brine (50 ml), dried over Na_2_SO_4_ and filtered. Upon standing, white crystals precipitated from the filtrate; these were dried under ambient conditions to give 510 mg of the product (**2**) (81%) as a white powder: ^1^H NMR (500 MHz, DMSO‐*d*_6_) δ ppm 12.15 (s, 1H), 8.94 (s, 1H), 8.71 (s, 1H), 8.48 (s, 1H), 7.63 (d, *J* = 3.5 Hz, 1H), 7.09 (d, *J* = 3.5 Hz, 1H), 4.61 (d, *J* = 9.5 Hz, 2H), 4.24 (d, *J* = 9.5 Hz, 2H), 3.70 (s, 2H); ^13^C NMR (126 MHz, DMSO‐*d*_6_) δ ppm 152.2, 150.9, 149.3, 139.9, 129.6, 126.9, 122.2, 116.6, 113.0, 99.9, 58.5, 56.0, 26.8. HRMS (ESI‐TOF) *m/z*: [M + H]^+^ calcd for C_16_H_12_D_5_N_7_O_2_S 377.15350; found 377.15510.

## CONFLICT OF INTERESTS

The authors report no conflicts of interest.

## Data Availability

Data are contained within the article.

## References

[1] Stebbing J , Phelan A , Griffin I , et al. COVID‐19: combining antiviral and anti‐inflammatory treatments. Lancet Infect Dis. 2020;20:400‐402. 32113509 10.1016/S1473-3099(20)30132-8 PMC7158903

[2] Marconi VC , Ramanan AV , de Bono S , et al. Efficacy and safety of baricitinib for the treatment of hospitalised adults with COVID‐19 (COV‐BARRIER): a randomised, double‐blind, parallel‐group, placebo‐controlled phase 3 trial. Lancet Respir Med. 2021;9:1407‐1418. 34480861 10.1016/S2213-2600(21)00331-3 PMC8409066

[3] Titanji BK , Farley MM , Mehta A , et al. Use of baricitinib in patients with moderate to severe coronavirus disease 2019. Clin Infect Dis. 2020;72:1247‐1250. 10.1093/cid/ciaa879 PMC7337637 32597466

[4] Jorgensen SCJ , Tse CLY , Burry L , Dresser LD . Baricitinib: a review of pharmacology, safety, and emerging clinical experience in COVID‐19. Pharmacotherapy. 2020;40:843‐856. 32542785 10.1002/phar.2438 PMC7323235

[5] Stebbing J , Nievas GS , Falcone M , et al. JAK inhibition reduces SARS‐CoV‐2 liver infectivity and modulates inflammatory responses to reduce morbidity and mortality. Sci Adv. 2021;7:eabe4724. 33187978 10.1126/sciadv.abe4724 PMC7775747

[6] Atzrodt J , Derdau V . Pd‐ and Pt‐catalyzed H/D exchange methods and their application for internal MS standard preparation from a Sanofi‐Aventis perspective. J Label Compd Radiopharm. 2010;53:674‐685.

[7] Burrell RC , Bonacorsi SJ Jr , Ortiz A , Benkovics T , Shi Z . Synthesis of carbon‐14 and stable isotope labeled Censavudine. J Label Compd Radiopharm. 2022;1‐11. 10.1002/jlcr.3964 35106813

[8] Tung RD . Deuterated baricitinib. US10842792B2; 2020.

[9] Xu J , Cai J , Chen J , et al. An efficient synthesis of baricitinib. J Chem Res. 2016;40(4):205‐208.

[10] Rodgers JD , Maduskuie SSTP , Wang H , et al. Heteroaryl substituted pyrrolo[2,3‐*b*]pyridines and pyrrolo[2,3‐*b*]pyrimidines as Janus kinase inhibitors. US20070135461; 2007.

[11] Chen G‐L , He S‐H , Cheng L , Liu F . Copper‐catalyzed N‐directed distal C(sp^3^)–H sulfonylation and thiolation with sulfinate salts. Org Lett. 2021;23:8338‐8342. 34632768 10.1021/acs.orglett.1c03075

[12] Yang Z , Zheng Y , Xu J . Simple synthesis of sulfonyl chlorides from thiol precursors and derivatives by NaClO_2_‐mediated oxidative chlorosulfonation. Synlett. 2013;24(16):2165‐2169.

[13] Huang Z , Xu J . One‐pot synthesis of symmetric 1,7‐dicarbonyl compounds via a tandem radical addition–elimination–addition reaction. RSC Adv. 2013;3:15114‐15120.

[14] Prakash GKS , Mathew T , Panja C , Olah GA . Chlorotrimethylsilane−nitrate salts as oxidants: direct oxidative conversion of thiols and disulfides to sulfonyl chlorides. J Org Chem. 2007;72:5847‐5850. 17585828 10.1021/jo070907g

[15] Blotny G . A new, mild preparation of sulfonyl chlorides. Tetrahedron Lett. 2003;44(7):1499‐1501.

[16] Ye L , Wu J , Chen W , Feng Y , Shen Z . Novel anti‐cancer agents based on germacrone: design, synthesis, biological activity, docking studies and MD simulations. RSC Adv. 2017;7:3760‐3767.

[17] Yang Z , Xu J . Convenient and environment‐friendly synthesis of sulfonyl chlorides from *S*‐alkylisothiourea salts via *N*‐chlorosuccinimide chlorosulfonation. Synthesis. 2013;45:1675‐1682.

[18] Park YJ , Shin HH , Kim YH . Convenient one‐pot synthesis of sulfonyl chlorides from thiols using sulfuryl chloride and metal nitrate. Chem Lett. 1992;21(8):1483‐1486.

[19] Mochel WE , Agre CL , Hanford WE . Interchange reactions of orthothioformates and mercaptoles. J Am Chem Soc. 1948;70:2268‐2269.

[20] Rodgers JD , Shepard S , Li Y‐L , Zhou J , Liu P , Meloni D , Xia M . Azetidine and cyclobutane derivatives as JAK inhibitors. WO2009114512 A1; 2009.

[21] Ireland RE , Varney MD . Approach to the total synthesis of chlorothricolide: synthesis of (.+‐.)‐19,20‐dihydro‐24‐O‐methylchlorothricolide methyl ester ethyl carbonate. J Org Chem. 1986;51:635‐648.

[22] Garg NK , Sarpong R , Stoltz BM . The first total synthesis of dragmacidin D. J Am Chem Soc. 2002;124:13179‐13184. 12405846 10.1021/ja027822b

[23] Friedman PA , Fridman JS , Luchi ME , Williams WV . Janus kinase inhibitors for treatment of dry eye and other eye related diseases. WO2010039939; 2010.

[24] Matthews DP , Whitten JP , McCarthy JR . Halogenation of [2‐(trimethylsilyl)ethoxy]methyl (SEM) protected 2,2′‐Bi‐*H*‐imidazole. J Heterocyclic Chem. 1987;24:689‐692.

[25] Chandra T , Broderick WE , Broderick JB . An efficient deprotection of *N*‐trimethylsilylethoxymethyl (SEM) groups from dinucleosides and dinucleotides. Nucleosides Nucleotides Nucleic Acids. 2010;29:132‐143. 20391200 10.1080/15257771003612847 PMC4331121

[26] Armarego WLF , Chai C . Purification of Laboratory Chemicals. 5thed. Elsevier; 2003.

[27] Langler RF . A facile synthesis of sulfonyl chlorides. Can J Chem. 1976;54:498‐499.

